# Anticonvulsant effect of *Satureja hortensis* aerial parts extracts in mice

**Published:** 2016

**Authors:** Farzaneh Zolfagharian, Bibi Marjan Razavi, Hossein Hosseinzadeh

**Affiliations:** 1 *School of Pharmacy, Mashhad University of Medical Sciences, Mashhad, Iran*; 2 *Targeted Drug Delivery Research Center, Department of Pharmacodynamy and Toxicology, School of Pharmacy, Mashhad University of Medical Sciences, Mashhad, Iran*; 3 *Pharmaceutical Research Center, Department of Pharmacodynamy and Toxicology, School of Pharmacy, Mashhad University of Medical Sciences, Mashhad, Iran*

**Keywords:** *Satureja hortensis*, *Seizure*, *Pentylenetetrazol*, *Maximal electroshock*, *Flumazenil*, *7-nitro- indazole*

## Abstract

**Objective:**

Regarding the anticonvulsant effects of *Satureja hortensis* (*S. hortensis*) in Avicenna’s book: canon of medicine; the present study was undertaken to evaluate the anti- eplileptic effects of *S. hortensis* aqueous and ethanolic aerial part extracts. Furthermore, the mechanisms of their anticonvulsant activities were also evaluated.

**Materials and Methods:**

Seizure was induced by Pentylentetrazol (PTZ) and MES (maximal electroshock) models. Mice were randomly divided into 8 groups; negative control (normal saline, 10ml/Kg), positive control (diazepam, 2 mg/kg), *S. hortensis *aqueous and ethanolic extracts (200, 400 and 600 mg/kg). In PTZ test, latency to the first minimal clonic seizure (MCS), latency to the first generalized tonic–clonic seizures (GTCS), the total duration of seizures and protection against mortality were evaluated. In MES test, the stretching length of extremities and protection against mortality were recorded.

**Results:**

Aqueous and ethanolic extracts (400 and 600 mg/kg) significantly increased MCS and GTCS latencies in PTZ model. Three doses of the extracts decreased the total duration of seizure. These extracts did not show any protective effects on seizure induced by MES model. In PTZ model, flumazenil, an antagonist of benzodiazepine (BZD) site in the GABA_A_-BZD receptor complex and 7- nitroindazole (7- NI), a selective nNOS (neuronal nitric oxide synthase) inhibitor, reduced the prolongation of seizure latency.

**Conclusion:**

*S. hortensis* showed anticonvulsant activity in PTZ model and this effect may be mediated, at least partly, through interacting with nitric oxide and GABA_A_-BZD receptor complex.

## Introduction

Epilepsy is defined as one of the most common serious neurological illness which is characterized by recurrent seizures (Porter et al., 2001). The incidence of epilepsy is about 0.5-1%. It can occur at any age (Hosseinzadeh et al., 2013). Although several anti-epileptic drugs are used to treat convulsions, due to the incomplete medication of about 30% of patients, side effects of these drugs, and chronic nature of epileptic disease, herbal medicines are widely recommended (Gorgich et al., 2012). Herbal medicines are being used for the treatment of a variety of disorders including neurological diseases because of their safety, efficacy, cultural acceptability and fewer side effects (Semnani et al., 2007). According to the literature, some plants and their active constituents including *Crocus sativus* (Hosseinzadeh et al., 2007),* Nigella sativa* (Hosseinzadeh et al., 2004), *Hypericum perforatum (*Hosseinzadeh et al., 2004)*,* *Justicia extensa* (Sowemimo et al., 2012), *Annona senegalensis (*Okoye et al., 2013), *Zyzyphus jujube* (Pahuja et al., 2012) , *Harpagophytum procumbens* (Mohamed et al., 2006), *Sutherlandia frutescens* (Ojewole et al., 2008), and *Zingiber officinale* (Hosseini et al., 2014), exhibit anticonvulsant activity. 

*Satureja hortensis *L. (*S. hortensis *L.) is a plant belongs to *Lamiaceae *family (*Labiatae*). It is distributed in the Europe, Asia and northern Africa. This plant is also cultivated in Iran (Fathi et al., 2013. Kamkar et al., 2013). Besides its use in cookery, *S. hortensis* is utilized for treating many diseases including cardiovascular diseases, gastrointestinal disorders, muscle pains, cramps, and infectious diseases in traditional and modern medicines. Moreover, different properties including antibacterial, antifungal, antioxidant, analgesic and carminative effects have been attributed to this plant (Fathi et al., 2013; Dorman et al., 2004; Yazdanparast et al., 2012). 

Since there are some reports in traditional medicine regarding the use of *S. hortensis* for the treatment of seizure (Asadi et al., 2012). The anticonvulsant activity of aqueous and ethanolic extracts of *S. hortensis* was evaluated in this study. 

## Materials and Methods

**Animals**

Male albino mice (weighing 25 ± 3 g) have been used in this study. Animals were housed in a ventilated room under a 12/12-hour light/dark cycle at 24 °C with free access to water and food. All animals in these experiments were carried out in accordance with Mashhad University of Medical Sciences, Ethical Committee Acts.

**Plant**

The aerial parts of *S. hortensis* were collected from Mashhad, and were identified by Mr. Joharchi and voucher samples were preserved for reference in the Herbarium of the Department of Pharmacognosy, School of Pharmacy, Mashhad (Voucher no. 1402).

**Preparation of extract**

*S. hortensis *aerial parts were cleaned, dried in shadow and powdered by a mechanical grinder. Then, the powder of leaves (100 g) was defatted with petroleum ether (40–60 °C) using the Soxhlet apparatus. For the ethanolic extract, the powder (100 g) was subsequently macerated in 1000 ml ethanol (70%, v/v) for 72 hours and the mixture was filtered and concentrated in vacuum at 40 °C. Then, to obtain dried powder, the residue was freeze dried. For the aqueous extract, 1000 ml distilled water was added to 100 g of aerial parts powder and was boiled for about 20 minutes. Then it was filtered. The extract was then concentrated in vacuum to the desired volume and then freeze dried.

**Study design**

The mice were randomly divided into 8 groups of six animals each: (1) negative control (normal saline, 10 ml/kg), (2) positive control (diazepam, 2 mg/kg), (3, 4, 5) ethanolic extract (200, 400, and 600 mg/kg, y) and (6, 7 and 8) aqueous extract (200,400 and 600 mg/kg). The selected doses of extracts were based on the calculated maximum tolerated dose in our pilot study.

**Anticonvulsant activity**

*Pentylenetetrazol (PTZ) induced seizure test*

Ethanolic extract, normal saline and diazepam were administrated intraperitoneally, 30 minutes prior to pentylenetetrazole (PTZ) (90 mg/Kg). The aqueous extract was administrated intraperitoneally, 60 minutes prior to PTZ (Hosseinzadeh et al., 2004). The animals were placed individually in plastic boxes and observed for 20 minutes.

In PTZ model, the latency to the first minimal clonic seizure (MCS), latency to the first generalized tonic–clonic seizures (GTCS), the total duration of seizures and protection against mortality were evaluated (Hosseinzadeh et al., 2000; Ramezani et al., 2004). In another experiment, flumazenil (10 mg/kg), an antagonist of benzodiazepine (BZD) site in the GABA_A_-BZD receptor complex (Hosseinzadeh et al., 2007) was administrated 30 minutes prior to the extracts. 7-NI (10 mg/kg), a selective nNOS inhibitor (Babbedge et al., 1993) was also administrated 60 minutes prior to *S.hortensis* L. extracts. 

*Maximal electroshock seizure (MES) test*

Ethanolic extract, normal saline and diazepam were administrated intraperitoneally, 30 minutes prior to the MES test. The aqueous extract was administrated intraperitoneally, 60 minutes prior to the MES test (Hosseinzadeh et al., 2004). Then, the stimulus train was applied via ear-clip electrodes (sinusoidal pulses, 120 mA and 60 Hz, for 0.2 seconds) using a constant current stimulator (Digital Electroshock Model 150, EghbalTeb Co., Mashhad, Iran). A drop of 0.9% saline solution was applied on each ear of the animal prior to placing the electrode. The duration of hind limb tonic extension (HLTE), and the protection against mortality were recorded (Hosseinzadeh et al., 2000). 

**Statistical analysis**

All results are expressed as mean±SEM. ANOVA followed by Tukey–Kramer test were performed to compare the means. p values less than 0.05 were considered as significant. 

## Results

**Anticonvulsant activity**

*PTZ-induced seizure test*

**Figure 1:**
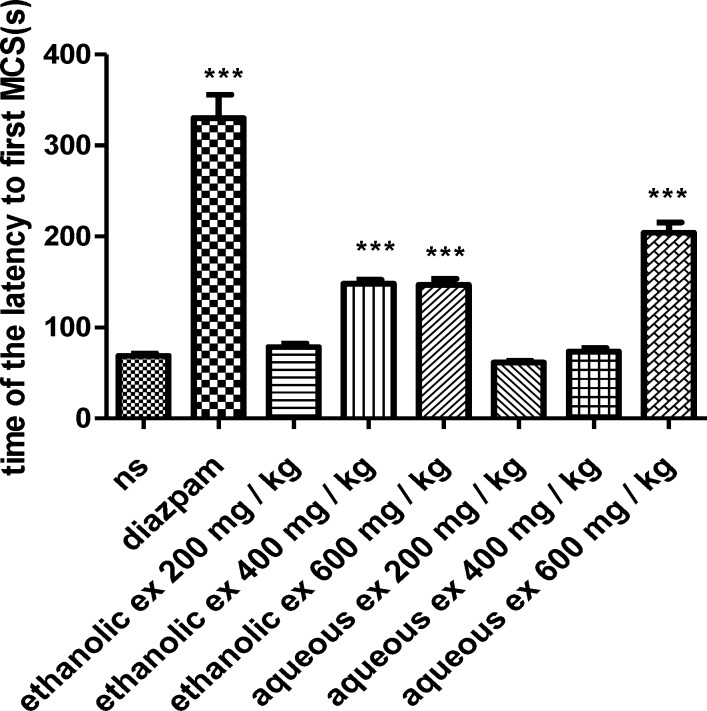
Effect of ethanolic and aqueous extracts of *S. hortensis *on the latency to the first minimal clonic seizure (MCS) in PTZ-induced seizure in mice. Data are presented as mean ± SEM. Tukey Kramer, p<00.1 vs normal saline, n=6. (ns= Normal saline , ex= Extract

**Figure 2:**
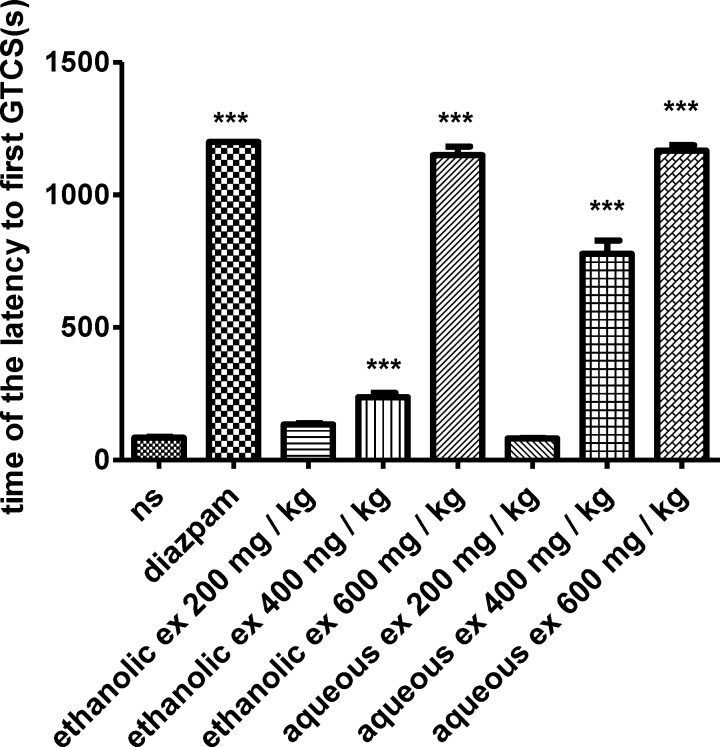
Effect of ethanolic and aqueous extracts of S. hortensis on the latency to first generalized tonic-clonic seizure (GTCS) in PTZ-induced seizure in mice. Data presented as mean ± SEM. Tukey Kramer, p<00.1 vs normal saline, n=6. (ns= Normal saline , ex= Extract

**Figure 3.**
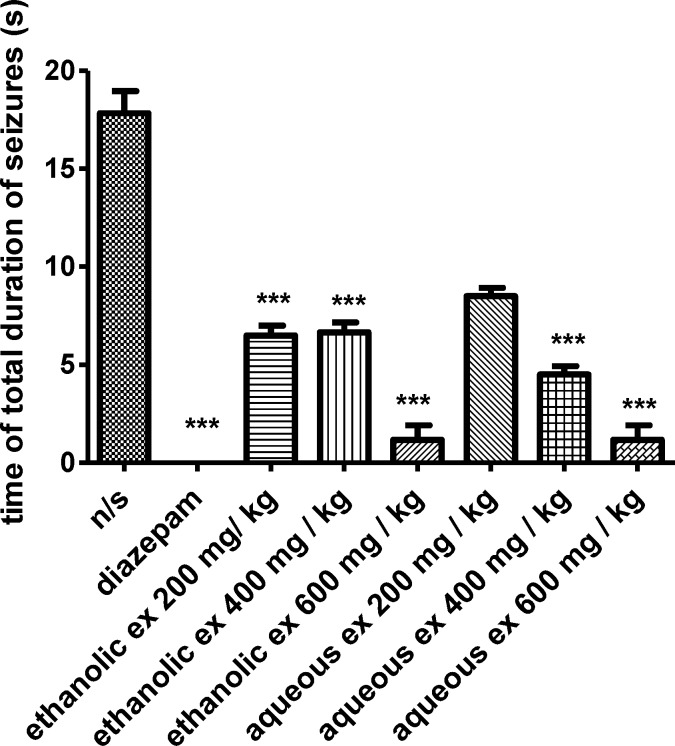
Effect of ethanolic and aqueous extracts of *S. hortensis *on total duration of seizures in PTZ-induced seizure in mice. Data presented as mean ± SEM. Tukey Kramer, p<00.1 vs normal saline, n=6. (ns= Normal saline , ex= Extract

In the PTZ-induced seizure, administration of the ethanolic extract (400 and 600 mg/kg) (p< 0.001), increased the latency to the first minimal clonic seizure (MCS) (Figure 1) and latency to the first generalized tonic–clonic seizures (GTCS) (Figure 2), compared to the negative control group. The ethanolic extract (200, 400 and 600 mg/kg) (p<0.001) decreased the total duration of seizure (Figure 3). The aqueous extract (600 mg/kg) increased MCS (Figure 1) and GTCS (Figure 2) and decreased total duration of seizure (400 and 600 mg/kg) (Figure 3). 

Moreover, the aqueous extract (600 mg/kg) protected against mortality after 30 minutes and 24 hours (p<0.05) (Table 1). 

**Table 1:** Effect of aqueous and ethanolic extracts of *S. hortensis *on mortality (%) in PTZ model after 30 minutes and 24 hours in mice. Fisher test. p<0.0 vs normal saline. n=6

**Agents**	**Protection against mortality (%) after 30 minutes**	**Protection against mortality (%) after 24 hours**
**Normal saline**	33	17
**Diazepam**	100^*^	100*
**Aqueous extract of ** ***S. hortensis *** **(200mg/kg)**	50	33
**Aqueous extract of ** ***S. hortensis *** **(400mg/kg)**	66	66
**Aqueous extract of ** ***S. hortensis *** **(600mg/kg)**	83*	83*
**Ethanolic extract of ** ***S. hortensis *** **(200mg/kg)**	50	50
**Ethanolic extract of ** ***S. hortensis *** **(400mg/kg)**	66	66
**Ethanolic extract of ** ***S. hortensis *** **(600mg/kg)**	83*	83*

*MES test*

**Figure 4:**
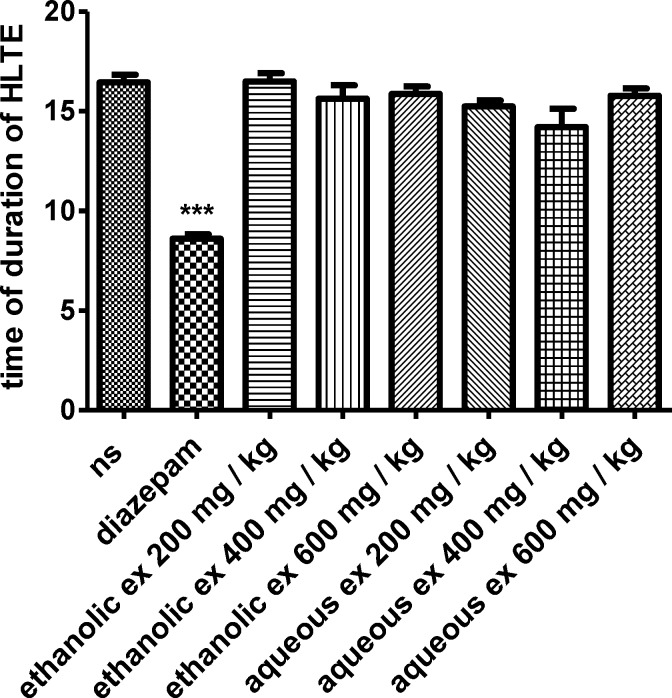
Effect of aqueous and ethanolic extracts of *S. hortensis *on duration of hind limbtonic extension (HLTE) in MES-induced seizure in mice. Data presented as mean ± SEM. Tukey Kramer, p<00.1 vs normal saline, n=6. (ns= Normal saline , ex= Extract

In the MES test, both extracts could not reduce the duration of hind limb tonic extension (HLTE) (Figure 4). Although the extract reduced the mortality (%) compared to the normal saline, this reduction was not statistically significant (Table 2).

**Table 2:** Effect of aqueous and ethanolic extracts of *S. hortensis *on mortality (%)in MES model in mice. Fisher test. p<0.0 vs normal saline. n=6

**Agents**	**Protection against mortality (%) **
**Normal saline**	16
**Diazepam**	100^*^
**Aqueous extract of ** ***S. hortensis *** **(200mg/kg)**	33
**Aqueous extract of ** ***S. hortensis *** **(400mg/kg)**	33
**Aqueous extract of ** ***S. hortensis *** **(600mg/kg)**	50
**Ethanolic extract of ** ***S. hortensis *** **(200mg/kg)**	33
**Ethanolic extract of ** ***S. hortensis *** **(400mg/kg)**	50
**Ethanolic extract of ** ***S. hortensis *** **(600mg/kg)**	66

**The effect of 7-NI on the anticonvulsant activity of** ***S. hortensis*** **extracts**

**Figure 5:**
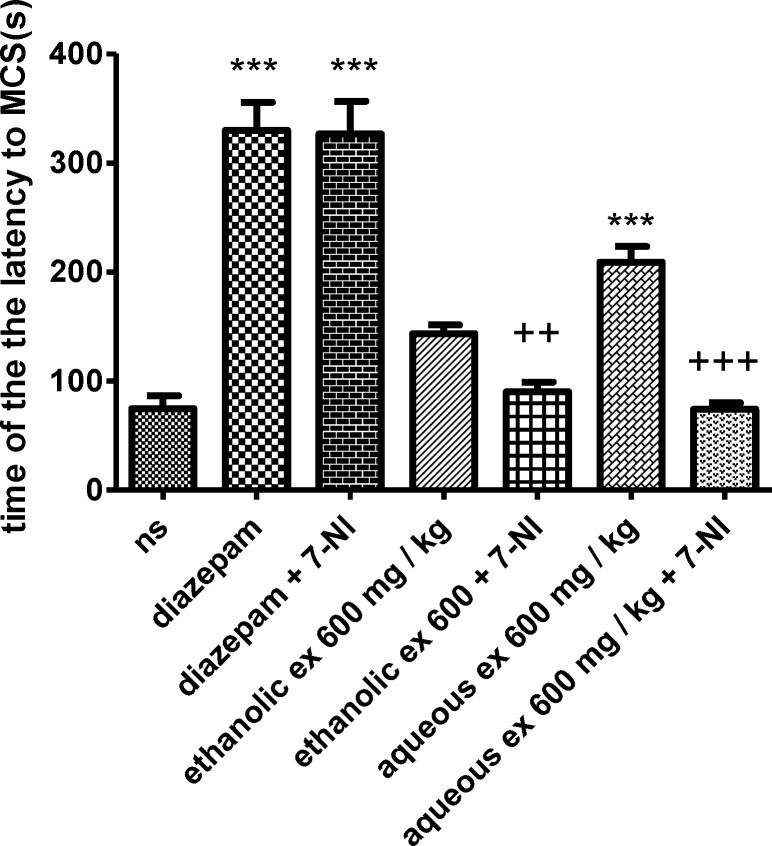
Effect of aqueous and ethanolic extracts of *S. hortensis *on the latency to first minimal clonic seizure (MCS) in PTZ-induced seizure in the presence and absence of 7-NI in mice. Data presented as mean ± SEM. Tukey Kramer, ^***^p<00.1 vs normal saline, ^+++^ p<0.001 and ^++^ p<0.01 vs extracts received 7- nitroindazol. n=6. (ns= Normal saline , ex= Extract , 7-NI=7- nitroindazole

**Figure 6:**
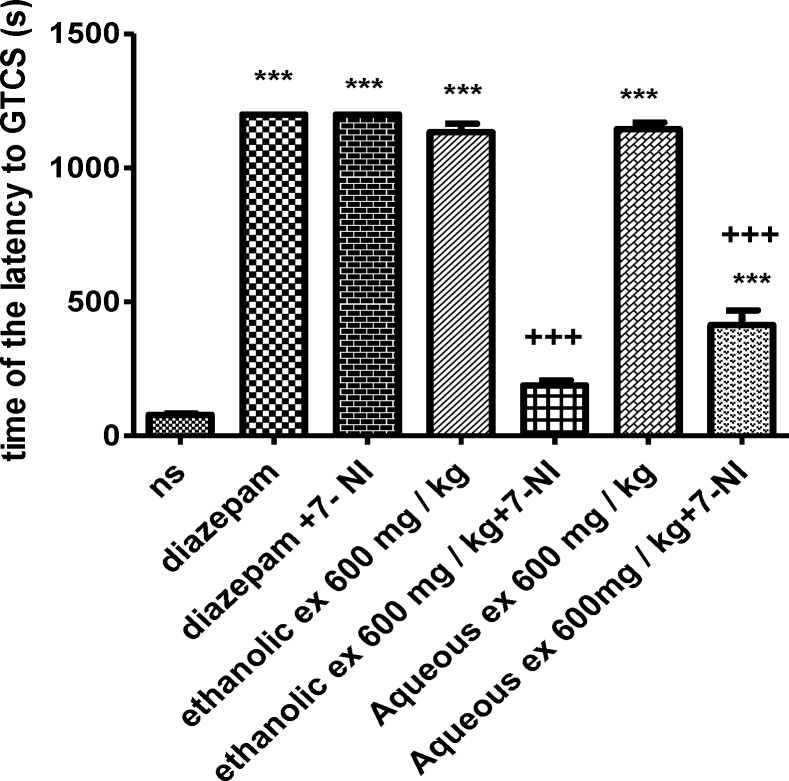
Effect of aqueous and ethanolic extracts of *S. hortensis *on the latency to the first generalized tonic-clonic seizure (GTCS) in PTZ-induced seizure in the presence and absence of 7- NI in mice. Data presented as mean ± SEM. Tukey Kramer, ^***^p<00.1 vs normal saline, +++ p<0.001 vs extracts received 7- nitroindazol. n=6. (ns= Normal saline , ex= Extract , 7-NI=7-NI

The results showed that 7-NI significantly decreased the anticonvulsant effect of the aqueous and ethanolic extracts (600 mg/kg) against PTZ-induced seizures by reducing the latency to the first minimal clonic seizure (MCS) (p<0.001 and 0.01, respectively) (Figure 5) and the latency to the first generalized tonic–clonic seizures (GTCS) (p<0.001) (Figure 6).

**The effect of flumazenil on the anticonvulsant activity of** ***S. hortensis*** **extracts**

**Figure 7:**
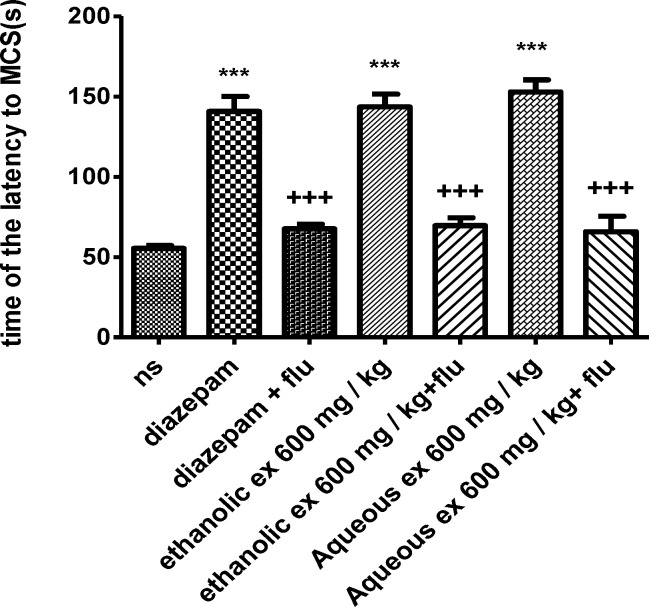
Effect of aqueous and ethanolic extracts of *S. hortensis *on the latency to first minimal clonic seizure (MCS) in PTZ-induced seizure in the presence and absence of flumazenil in mice. Data presented as mean ± SEM. Tukey Kramer, ^***^p<00.1 vs normal saline, ^+++^ p<0.001 vs extracts received flumazenil. n=6. (ns= Normal saline , ex= Extract , flu= Flumazenil

**Figure 8:**
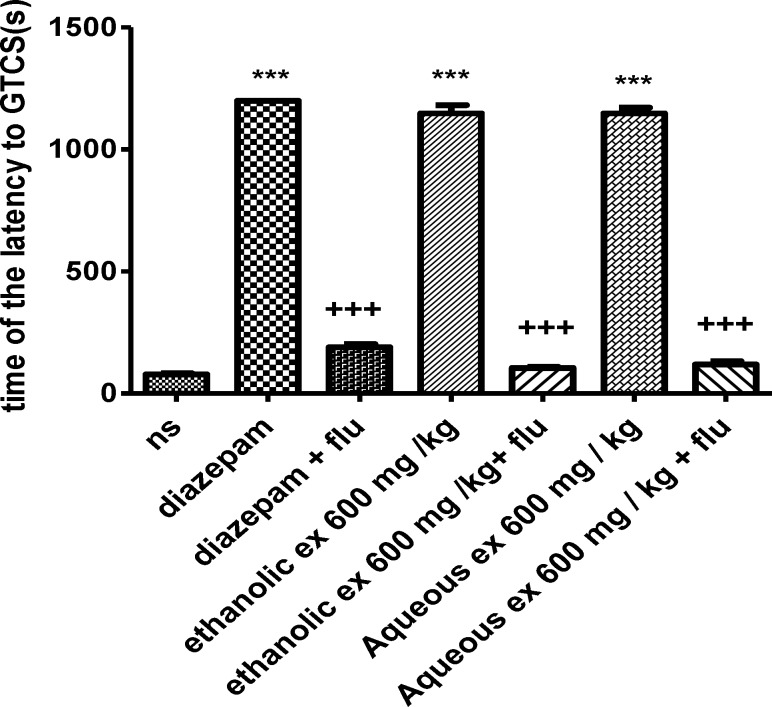
Effect of aqueous and ethanolic extracts of *S. hortensis *on the latency to first generalized tonic-clonic seizure (GTCS) in PTZ-induced seizure in the presence and absence of flumazenil in mice. Data presented as mean ± SEM. Tukey Kramer, ^***^p<00.1 vs normal saline, ^+++^ p<0.001 vs extracts received flumazenil. n=6. (ns= Normal saline , ex= Extract , flu= Flumazenil

Flumazenil reduced the anticonvulsant activity of the aqueous and ethanolic extracts (600 mg/kg) by decreasing the latency to the first minimal clonic seizure (MCS) (p<0.001) (Figure 7) and the latency to the first generalized tonic–clonic seizures (GTCS) (p<0.001) (Figure 8).

## Discussion

This study indicated that ethanolic and aqueous extracts of *S. hortensis *exhibited anticonvulsant effects in the PTZ-induced seizure model but not in the MES induced seizure model. These extracts increased the latency to first minimal clonic seizure (MCS) and the latency to the first generalized tonic–clonic seizures (GTCS) and decreased the total duration of seizure compared to the negative control group especially at higher doses. Furthermore, pretreatment with flumazenil (10 mg/kg) or 7-NI (10 mg/kg), prior to extracts (600 mg/kg, i.p.), reduced the protective effect of *S. hortensis *against PTZ-induced seizure. 

PTZ, is a chemical which blocks selectively the chloride channel coupled to the GABA_A_ receptor complex (Seima et al., 1997). According to the documents, PTZ- induced seizure model could be used for the evaluation of absence epilepsy, so agents with anticonvulsant effects in the absence epilepsy are effective in PTZ-induced seizure model (Porter et al., 1984). Based on the data, *S. hortensis *extracts displayed anticonvulsant activity in the absence epilepsy.

Maximal electroshock (MES) induced seizure is a model to evaluate anticonvulsant properties of compounds that affect the tonic clonic epilepsy.^[26]^ Neither ethanolic nor aqueous extracts of *S. hortensis *reduced the duration of hind limb tonic extension. Therefore, this plant was not effective in the tonic clonic epilepsy. It is also indicated that compounds which can inhibit voltage dependent sodium channels, show anticonvulsant properties in the MES induced seizure (Aquair et al., 2012). So, according to our data, this plant may not have any effect on the sodium channels.

It is found that changing in some neurotransmitter systems such as the glycine, glutamatergic, GABAergic and some molecules like nitric oxide could be considered as potential mechanisms involved in the induction of epilepsy (Engelborghs et al., 2000; Ure et al., 2000). 

7-NI is considered as a selective inhibitor of neuronal NOS (nNOS) (Babbedge et al., 1993). It is established that 7-NI can abolish the anticonvulsant effects of agomelanine in PTZ- induced seizure model. Dastgheib et al (2014) concluded that agomelanine exerts its anti-epileptic effect partly due to the nNOS induction (Dastgheib et al., 2014). In our study, 7-NI was administrated 60 minutes prior to the extracts. Since 7-NI decreased the protective effect of *S. hortensis *against PTZ-induced seizure, its anticonvulsant effects may be attributed to the interaction with nitric oxide pathway.

Flumazenil is an antagonist of GABA_A_-benzodiazepine receptor (Hosseinzadeh et al., 2007). Administration of flumazenil 30 minutes prior to the extracts significantly decreased their anticonvulsant activity, so it might be concluded that this plant shows anticonvulsant effect through GABA_A_-benzodiazepine receptor complex.

It is documented that the effect of flumazenil (10 mg/kg) or 7-NI (10 mg/kg) alone on PTZ-induced seizure is similar to that of the control group (Borowicz et al., 2000). Based on these results and limitations of cost and time, the effect of these chemicals alone on PTZ-induced seizure was not evaluated in our study.

Our results also demonstrated that the ethanolic extract was likely as effective as the aqueous extract of *S. hortensis *in the absence epilepsy; suggesting that its effective constituents may be polar but some semi and non polar constituents may also be present. 

There have been reports that monoterpenoid phenols such as carvacrol show anticonvulasant activity in PTZ and MES induced seizure models through interacting with GABA _A_ receptor (Lucindo et al., 2010).

Carvacrol is abundantly found in the essential oils of the lamiaceae family (Lucindo et al., 2010). So it may be concluded that the protective effect of this plant on PTZ-induced seizures is at least partly due to the presence of these constituents, but further investigations to explore the main constituents required for the anticonvulsant activity of this plant are recommended.

This study indicated that *S. hortensis *could exert anticonvulsant activity in the PTZ model and this effect may be mediated, at least partly, through interaction with nitric oxide and GABA_A_-BZD receptor complex. 
